# Social anxiety and social networking service addiction proneness among university students: A moderated mediation model of narcissism and gender

**DOI:** 10.1371/journal.pone.0304741

**Published:** 2024-06-03

**Authors:** Jin Pyo Lee

**Affiliations:** 1 Department of Nursing Science, U1 University, Yeongdong-gun, Chungbuk, Republic of Korea; 2 Department of Medicine, College of Medicine, Chungbuk National University, Cheongju, Republic of Korea; National University of Medical Sciences, PAKISTAN

## Abstract

This study aims to investigate the mediation effect of narcissism and the mediation effect moderated by gender in the effect of social anxiety on university students’ SNS addiction. In this cross-sectional survey, university students, aged 19 to 29 were selected from two provinces in South Korea. The sample size was calculated using G*power 3.1., and a sample of 170 university students was used in the final analysis. To perform the analysis, descriptive statistics; independent t-test, one-way ANOVA, and Pearson’s correlation were used. The data collected was statistically analyzed using SPSS Program 23.0 and SPSS PROCESS macro (version 4.0). The moderated mediation effect was significant in both male and female groups. The mediation effect of narcissism on the relationship between social anxiety and SNS addiction proneness was stronger in the female group than in the male group. The findings have the potential to provide substantial basic data for developing health promotion and education programs to reduce university students’ social anxiety, narcissism, and SNS addiction.

## Introduction

Social networking services (SNS) have become another social space for maintaining or establishing new relationships with others without time or space constraints. SNS is a major communication tool in the information society, a virtual community where users can create personal profiles and interact with others [1]. The widespread use of smartphones has also increased the accessibility to SNS. SNS provides access to various features to the users such as communicating with people, sharing information, and keeping records of their lives [2]. Similarly, the platform provided by SNS enables users to form intimacy with others and maintain interpersonal relationships [3]. Among students, the rate of using SNS is higher in university students (93.5%) than in higher school (77.4%) or middle school students (74.4%) [4]. The high rate of use of SNS among university students is attributed to their familiarity with smart devices, sensitivity to friendships or reactions from friends, and a higher sense of value for social relations [5].

Nevertheless, the rapid increase in the use of SNS has become a major concern as it causes SNS addiction [6]. In South Korea, ‘SNS addiction proneness’ is mostly used in place of SNS addiction, as a clear concept or diagnostic criteria for SNS addiction has not been established [7]. SNS addiction proneness refers to the act of withdrawal and tolerance as well as disruptions in daily life by being overly immersed in interpersonal relationships online [8]. SNS addiction proneness has been reported as the cause of various social problems experienced by SNS users such as relative deprivation, pretentious expressions about unwanted interactions, and hostility [9]. Moreover, it is one of the causes of emotional problems such as depression and anxiety among SNS users [10]. The problem of SNS addiction proneness is predominant among young adults [4]. However, as university students are in emerging adulthood and their identities are developed by interacting with others, superficial communication with others on SNS may hinder them from achieving their developmental goals [2,10,11]. Therefore, SNS addiction proneness has some negative effects on the daily lives of university students.

University students often feel anxiety about being evaluated or receiving negative evaluations by others. Social anxiety, marked by a fear of anxiety, typically manifests as fear or apprehension about interacting with others in social situations, which can occur during routine social interactions. Individuals may fear judgment or evaluation by others, or anticipate failure or rejection in interpersonal relationships [6,12]. Those who have high social anxiety are likely to be extremely nervous or feel anxiety and fear when having a conversation with others or in certain situations [12]. Those with high social anxiety are more active on SNS as it enables them to freely assert and express themselves, which increases SNS addiction proneness [13].

Furthermore, one of the psychosocial risk factors for SNS addiction proneness is narcissism. SNS users highly tend to reveal themselves positively and want to be acknowledged. Narcissists are characterized by an exaggerated sense of self-importance and tend to demand excessive flattery and an ostentatious attitude [14]. As highly narcissistic people are overly sensitive to being rejected or hurt, they may seem faint-hearted and timid and yet they internally have narcissism traits [15]. Narcissists tend to respond sensitively to other people’s reactions, and highly narcissistic people are more likely to excessively use SNS or be addicted to it [14,15]. Highly narcissistic people tend to be more active on Facebook or other forms of SNS to present a more positive image of themselves online [14,15].

The relationship between social anxiety and SNS addiction proneness varies depending on gender. Women showed significantly higher social anxiety than men, and they also showed higher SNS addiction proneness than men as well [5]. Moreover, in a study that analyzed the effect of social anxiety in university students on SNS addiction proneness, social anxiety affected SNS addiction proneness in male students, whereas no significant effect was found in female students [6]. Compared to real life, fewer factors increase social anxiety online, such as SNS, as well as greater anxiety control, which is why people experiencing social anxiety tend to rely on SNS [5]. This may also be related to the fact that male university students have a remarkably lower risk of negative evaluation of themselves and use SNS to form new relationships, whereas female students use SNS to maintain existing relationships [16]. Furthermore, highly narcissistic people are likely to use SNS excessively or be addicted to it. SNS addiction proneness is an issue that has been emphasized since the penetration of SNS [14,15]. While moderate use of SNS greatly helps with sharing information and forming interpersonal relationships, excessive use interferes with daily life and studies and leads to negative effects such as stress, depression, and anxiety [6,15]. This implies that the strength of the mediation effect of narcissism in the effect of social anxiety on SNS addiction proneness may vary depending on gender.

Accordingly, this study explores the variables affecting SNS addiction proneness and comprehensively examines the mediation effect of narcissism and the moderated mediation effect of gender in the effect of social anxiety on SNS addiction of university students. In addition, considering that gender may moderate the effect of social anxiety on SNS addiction proneness, this study explores the structural relationship of how social anxiety affects SNS addiction proneness through narcissism and examines whether this relationship may vary depending on gender. Based on the above, this study has the potential to provide substantial basic data for developing health promotion and education programs to reduce university students’ social anxiety, narcissism, and SNS addiction.

### Conceptual model and research purpose

**Fig 1:**
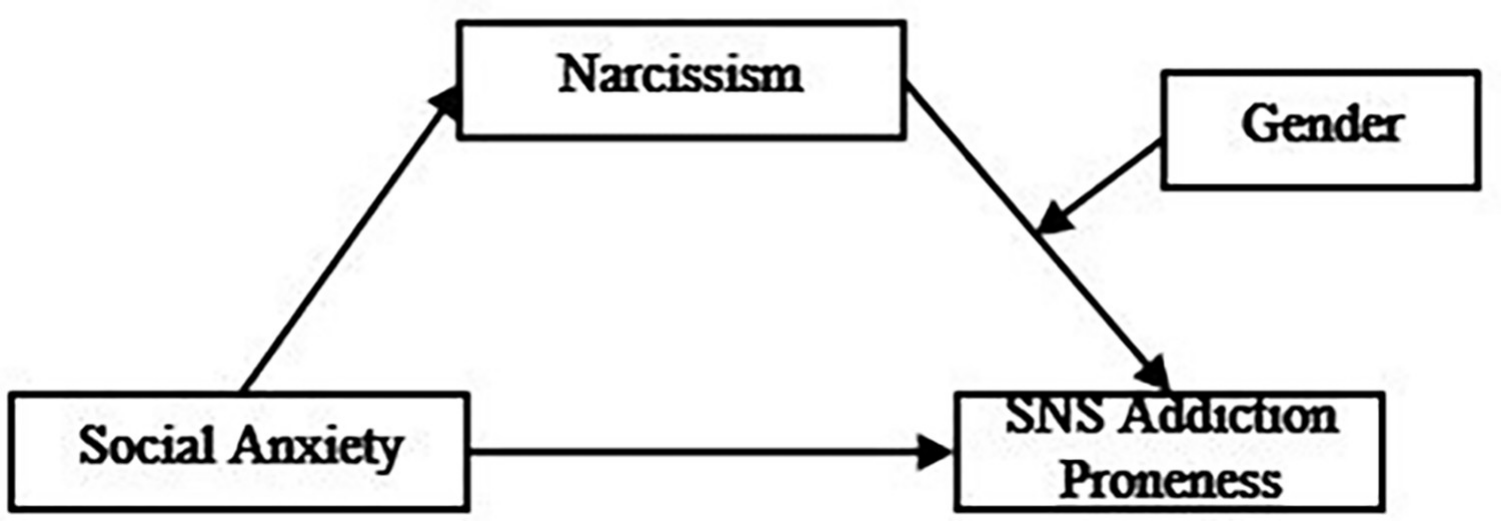
Conceptual framework of moderated mediation model.

The purpose of this study is to develop and validate a model that explains the mediation effect of narcissism and the mediation effect moderated by gender on the effect of social anxiety on university students’ SNS addiction. A summary of the literature review showed that both social anxiety and narcissism affect SNS addiction [5,6,13–15]. Moreover, narcissism affected SNS addiction [14,15] and had a mediation effect on the relationship between social anxiety and SNS addiction [6]. Gender is a key variable that influences social anxiety and narcissism, as well as SNS addiction [5,6,16]. Based on these previous studies, we developed a conceptual model as follows, suggesting that there will be a mediation effect moderated by gender in the process where social anxiety of university students affects SNS addiction proneness with narcissism as a mediator **(Fig 1)**.

- First, the social anxiety of the university students will have a positive effect on their SNS addiction proneness.
- Second, the mediation effect of narcissism: Higher social anxiety will lead to higher narcissism, and therefore to a higher level of SNS addiction proneness.
- Third, moderated mediation effect of gender: Gender will serve as a reinforcing factor in the positive indirect effect of social anxiety on SNS addiction proneness through narcissism.

## Method

### Research design

This cross-sectional study is conducted to investigate the mediation effect of narcissism and the mediation effect moderated by gender in the effect of social anxiety on university students’ SNS addiction proneness.

### Participants and data collection

#### Participants

The participants of this study, aged 19 to 29 attending 4 four-year universities in two provinces in South Korea, were selected through a convenience sampling technique. The sample size was calculated using G*power 3.1.9.7. The minimum sample size required for multiple regression analysis was 99 based on a power of 90%, a significance level (α) of.05, effect size of 0.15, and 9 predictor variables (research variables, participants’ characteristics). Based on the above, 190 copies of the questionnaire were distributed, considering the dropout rate, and 170 were retrieved and used in the final analysis. The survey was conducted on students who voluntarily agreed to participate in the study after they were provided with information on the research purpose and content, the rights of the participants, and the time required.

### Measures

#### Social anxiety

This study used the Korean Social Interaction Anxiety Scale (K-SIAS) and the Korean Social Phobia Scale (K-SPS). The Korean version of the Social Interaction Anxiety Scale (SIAS) and the Korean Social Phobia Scale (K-SPS) developed by Mattick and Clarke (1998) [17] and adapted and validated by Kim (2001) [18]. The social interaction anxiety scale consists of items measuring emotional, cognitive, and behavioral responses in various situations where social interaction is required. The social phobia scale consists of items measuring anxiety experienced in social situations where one is being observed by others or when the attention of others is present [17,18]. The Social Interaction Anxiety Scale had a total of 19 items, the Korean Social Phobia Scale had a total of 20 items, each of which was rated on a 5-point Likert scale of “Strongly disagree” (1 point), “Disagree” (2 points), “Neutral” (3 points), “Agree” (4 points), and “Strongly agree” (5 points). The total scores of both scales were utilized. Higher scores indicate higher levels of anxiety in social interaction and performance situations. The scores with higher scores indicating higher social anxiety. The reliability of the SIAS in the study by Kim (2001) was Cronbach’s α = .92 [18]. Cronbach’s α in this study is .92. The reliability of the SPS in the study by Kim (2001) was Cronbach’s α = .92. Cronbach’s α in this study is .93.

#### SNS addiction proneness

This study used the SNS addiction proneness scale for university students validated and used by Jeong (2014) to analyze university students [19]. The scale had a total of 24 items, each of which was rated on a 4-point Likert scale of “Strongly disagree” (1 point), “Somewhat disagree” (2 points), “Somewhat agree” (3 points), and “Strongly agree” (4 points). The scores ranged from 24 to 96, with higher scores indicating higher SNS addiction proneness. Cronbach’s α in Jeong’s (2014) study was .92 [19]. Cronbach’s α in this study is .94.

#### Narcissistic personality disorder

This study used the narcissistic personality disorder scale developed as a self-report inventory to measure personality disorder by Hwang (1995) [20], targeting non-clinical populations, supplementing the scale based on the diagnostic criteria in the Diagnostic and Statistical Manual of Mental Disorders (DSM-Ⅲ). The scale had a total of 18 items, each of which was rated on a 4-point Likert scale of “Strongly disagree” (1 point), “Somewhat disagree” (2 points), “Somewhat agree” (3 points), and “Strongly agree” (4 points). A higher score indicates a stronger narcissistic personality. The reliability of the narcissistic personality disorder in Hwang’s study was Cronbach’s α = .63 [20], and the Cronbach’s α in this study is .91.

### Data collection method and ethical considerations

This study was conducted after obtaining approval from the Institutional Review Board (IRB No. U1IRB2023-05) of the researcher’s affiliated institution. Data was collected from June 02 to June 20, 2023, using a non-face-to-face self-report questionnaire. The participants were to read the research descriptions and consent form and agree to participate, and the survey was designed to end automatically if they did not agree. The survey was conducted only on those who agreed to participate in the research. The questionnaire included the purpose and method of research, anonymity and confidentiality regarding participation, and the choice to agree or withdraw from participation, as well as the confirmation that the survey will not be used for purposes other than research.

### Data analysis method

The data collected was statistically analyzed using SPSS Program 23.0 and SPSS PROCESS macro (version 4.0). The characteristics of the participants and variables were analyzed using descriptive statistics; the differences in SNS addiction according to the characteristics of the participants were analyzed using an independent t-test and one-way ANOVA, and the post-hoc test was conducted using Duncan’s test. The correlation between social anxiety, narcissism, and SNS addiction was analyzed using Pearson’s correlation.

The mediation effect of narcissism in the relationship between social anxiety and SNS addiction was analyzed using PROCESS Macro Model 4, after which the significance of the mediation effect (indirect effect) was determined. The moderated mediation effect of gender on the mediation effect of narcissism in the relationship between social anxiety and SNS addiction proneness was analyzed using PROCESS Macro Model 14. The moderated mediation effect indicates that the strength of the mediation effect is moderated by the moderator variable (W) in the effect that the independent variable (X) has on the dependent variable (Y) through the mediator variable (M), meaning that there is a moderated mediation effect when the index of moderated mediation is significant [21]. The significance of the indirect effect, the conditional indirect effect, and the index of moderated mediation were tested based on the lower and upper limits estimated by analyzing the 95% confidence interval (bootstrapping 95% confidence interval [CI]) using bootstrapping (5,000 resamples) [22].

## Results

### The difference test of the characteristics of respondents of each variable

Overall, 40.0% of the participants were male and 60.0% were female; 10.6% were freshmen, 16.5% were sophomores, 12.9% were juniors, and 60.0% were seniors. For the number of SNS platforms used, 61.2% used 1, 25.9% used 2, and 12.9% used 3 or more platforms. As for the average time spent using SNS per day, 18.8% used for less than 30 minutes, 15.3% for 30 minutes to less than 1 hour, 23.5% for 1 hour to less than 2 hours, 22.4% for 2 hours to less than 3 hours, and 20.0% for 3 hours or more. For personal connections on SNS (friends, followers, group members), 17.6% of the participants had around 10, 9.4% had around 30, 8.2% had around 50, and 64.7% had around 100 personal connections. Among friends on SNS, the percentage of those whom the participants do not know or have never met offline (in real life) was 70% for 7.1% of the participants, 50% for 11.8%, 30% for 31.8%, and none for 49.4%. As for how much importance SNS has in daily life, 17.6% responded that there is very little, 36.5% that it is slightly necessary, 22.4% neutral, 18.8% relatively important, and 4.7% absolutely necessary **(Table 1)**.

**Table 1:** The difference test of the characteristics of respondents of each variable. (N = 170).

Variables	Categories	N(%)	Social anxiety		Narcissism		SNS addiction proneness	
			M±SD	t or F(*p*) post-hoc†	M±SD	t or F(*p*) post-hoc†	M±SD	t or F(*p*) post-hoc†
Gender	Male	68(40.0)	3.43±.60	1.920 (.057)	2.16±.73	-1.568 (.119)	1.98±.57	-2.835 (.005)
	Female	102(60.0)	3.26±.55		2.34±.78		2.24±.57	
Academic year	Freshman	18(10.6)	2.14±.76	.744 (.527)	1.91±.64	1.408 (.242)	1.60±.60	-2.835 (.214)
	Sophomore	28(16.5)	2.45±.94		2.27±.57		1.89±.48	
	Junior	22(12.9)	2.21±.77		2.13±.51		1.72±.52	
	Senior	102(60.0)	2.24±.70		2.15±.59		1.90±.67	
Number of SNS platforms used	1 ^a^	104(61.2)	2.27±.83	.389 (.678)	2.05±.58	2.945 (.055)	1.74±.61	7.064 (.001) a,b<c
	2 ^b^	44(25.9)	2.20±.58		2.23±.61		1.86±.53	
	≥3 ^c^	22(12.9)	2.38±.71		2.33±.48		2.27±.64	
SNS using time(an average day)	None & < 30min ^a^	32(18.8)	2.29±.87	2.317 (.059)	2.09±.49	1.932 (.107)	1.38±.54	20.794 (< .001) a,b<c,d<e
	30min-1 hour ^b^	26(15.3)	1.86±.53		2.06±.57		1.47±.30	
	1–2 hours ^C^	40(23.5)	2.41±.83		2.03±.59		1.86±.55	
	2–3 hours ^d^	38(22.4)	2.33±.66		2.14±.56		1.96±.57	
	≥3 hours ^e^	34(20.0)	2.29±.75		2.38±.65		2.40±.46	
Personal connections on SNS (friends, followers, group members)	About 10 people ^a^	30(17.6)	2.36±.83	1.465 (.226)	1.89±.56	4.434 (.005) a,b<c,d	1.53±.44	3.650 (.014) a<b,c,d
	About 30 people ^b^	16(9.4)	2.53±1.1		1.97±.65		1.77±.70	
	About 50 people^**C**^	14(8.2)	1.99±.63		2.48±.66		1.86±.82	
	About 100 people ^d^	110(64.7)	2.23±.68		2.19±.54		1.93±.59	
The ratio of interpersonal relations on SNS(never met offline)	70%	12(7.1)	2.08±.70	2.699 (.047) a,c,d<b	2.19±.93	1.365 (.225)	1.84±.72	1.744 (.160)
	50%	20(11.8)	2.68±1.03		2.34±.49		2.11±.53	
	30%	54(31.8)	2.2±.78		2.18±.53		1.74±.56	
	None	84(49.4)	2.17±.65		2.06±.58		1.84±.65	
As for how much importance SNS has in daily life	Very little ^a^	30(17.6)	2.47±.86	1.464 (.215)	2.19±.58	2.062 (.088)	1.45±.55	13.210 (< .001) a<b,c<d<e
	Slightly Necessary ^b^	62(36.5)	2.13±.72		2.04±.48		1.74±.50	
	Neutral ^c^	38(22.4)	2.36±.83		2.10±.64		1.81±.59	
	Relatively important ^d^	32(18.8)	2.15±.64		2.23±.61		2.24±.51	
	Absolutely necessary ^e^	8(4.7)	2.44±.60		2.61±.81		2.67±.74	

M = Mean; SD = Standard deviation.

† Post-hoc: Duncan.

For the difference in social anxiety depending on the general characteristics of the participants, it was found that the difference was significant depending on the percentage of those whom the participants do not know or have never met offline (in real life) among friends on SNS (p = .047). The result of the post-hoc analysis showed that those who responded at 50% had higher social anxiety than those who responded at 70%, 30%, and none **(Table 1)**.

For the difference in narcissism depending on the participants’ general characteristics, it was found that the difference was significant depending on personal connections on SNS (friends, followers, group members) (p = .005). The result of post-hoc analysis showed that those with around 50 or more personal connections on SNS (friends, followers, group members) had higher levels of narcissism than those with around 30 or fewer personal connections (p = .006) **(Table 1)**.

For the difference in SNS addiction proneness depending on the general characteristics of the participants, it was found that the difference was significant depending on gender (p = .005), the number of SNS platforms used (p = .001), the average time spent using SNS per day (p < .001), personal connections on SNS (p = .014), and how much importance SNS has in daily life. The result of post-hoc analysis showed that those who use more SNS platforms and spend more time using SNS per day also had high SNS addiction proneness. Those with around 30–100 personal connections on SNS showed higher SNS addiction proneness than those with around 10. SNS addiction proneness was highest among those who responded that it is absolutely necessary, followed by those who responded neutral and those who responded that SNS has very little importance in daily life (*p* < .001) **(Table 1)**.

### Correlation between social anxiety, narcissism, and SNS addiction proneness

As a result of analyzing the correlation between social anxiety, narcissism, and SNS addiction proneness, it was found that social anxiety had a significant positive correlation with narcissism (r = .18, *p* < .05) and SNS addiction proneness (r = .34, *p* < .001). Narcissism (r = .60, *p* < .001) had a significant positive correlation with SNS addiction proneness **(Table 2)**.

**Table 2:** Means, standard deviations, and correlation among social anxiety, narcissism, SNS addiction proneness. (N = 170).

Variables	M	SD	Social anxiety r(*p*)	Narcissism r(*p*)	SNS addiction proneness r(*p*)
Social anxiety	2.26	0.76	1		
Narcissism	2.14	0.58	.18 (< .05)	1	
SNS addiction proneness	1.84	0.62	.34 (< .001)	.60 (< .001)	1

### The mediation effect of narcissism in the effect of social anxiety of university students on SNS addiction proneness

After adjusting for characteristics variables, including the number of SNS platforms used, the average daily time spent on SNS, personal connections on SNS, and the perceived importance of SNS in daily life, which showed significant differences in SNS addiction proneness. The mediation effect of narcissism in the effect of social anxiety on university students’ SNS addiction proneness was analyzed using Process macro-4, and the statistical significance of the indirect effect was analyzed using bootstrapping. Social anxiety (independent variable, X) had a significant effect on SNS addiction proneness (dependent variable, Y) in Step 1 (B = 0.28, p < .001), and social anxiety (independent variable, X) had a significant effect on narcissism (mediator variable, M) in Step 2 (B = 0.14, p = .016). With social anxiety (X) included in Step 3, the direct effect of narcissism (M) on SNS addiction proneness (Y) was significant (B = 0.59, p < .001), and the mediation effect (indirect effect) of social anxiety (X) on SNS addiction proneness (Y) through narcissism (M) was also significant (B = 0.19, p < .001). The mediation effect of narcissism in the effect of social anxiety on SNS addiction proneness was .08, not including O at the 95% confidence interval, thereby proving to be significant **(Table 3)**.

**Table 3:** Mediating effect of narcissism between social anxiety and SNS addiction proneness and moderated mediation effect analysis of gender. (N = 170).

Model	Independent variable	Dependent variable	Coeff.	SE	t	*p* -value		R^2^	F		*p* -value
Mediating effect											
Step 1.	Social anxiety	SNS addiction proneness	0.28	0.06	4.68	< .001		0.12	21.94	< .001	
Step 2.	Social anxiety	Narcissism	0.14	0.06	2.44	.016		0.15	5.95	.016	
Step 3.	Social anxiety, Narcissism	SNS addiction proneness						0.41	59.77	< .001	
	Social anxiety	SNS addiction proneness	0.19	0.05	3.93	< .001					
	Narcissism	SNS addiction proneness	0.59	0.06	9.29	< .001					
					Effect		Boot. SE		Boot. 95% CI (LLCI~ULCI)		
Indirect effect (Social anxiety → Narcissism → SNS addiction proneness)					.084		.034		.02 ~.15		
Model	Independent variable	Dependent variable	Coeff.	SE	t	p -value		R^2^	F	p -value	
Moderated mediation effect											
Step 1.	Social anxiety	Narcissism	0.14	0.06	2.44	.016		0.03	5.951	.016	
Step 2.	Social anxiety	SNS addiction proneness	0.21	0.05	4.37	< .001		0.47	36.45	< .001	
	Narcissism	SNS addiction proneness	-0.01	0.22	-.04	.972					
	Gender	SNS addiction proneness	0.23	0.07	3.17	.002					
Narcissism × Gender		SNS addiction proneness	0.35	0.13	2.67	.008					
			Gender			Effect	Boot. SE		Boot. 95% CI (LLCI~ULCI)		
Conditional indirect effect of Social Anxiety on SNS Addiction according to values of the moderator(gender) (Social anxiety → Narcissism → SNS addiction proneness)			Mediator Narcissism, male			0.34	.10		.13 ~ .54		
			Mediator Narcissism, female			0.69	.08		.53 ~ .84		

Coeff. = Coefficient; SE = Standard error; Boot. = Bootstrapping; CI = Confidence interval; LLCI = Lower limit confidence interval; ULCI = Upper limit confidence interval; SD = Standard deviation.

### The moderated mediation effect of gender in the effect of social anxiety of university students on SNS addiction proneness through narcissism

After adjustment for the number of SNS platforms used, the average time spent using SNS per day, personal connections on SNS, and how much importance SNS has in daily life, which had significant differences in SNS addiction proneness. The moderated mediation effect of gender in the effect of social anxiety on SNS addiction proneness through narcissism was analyzed using PROCESS Macro Model 14, and the statistical significance of the conditional indirect effect of gender and the index of moderated mediation were analyzed using bootstrapping.

**Fig 2:**
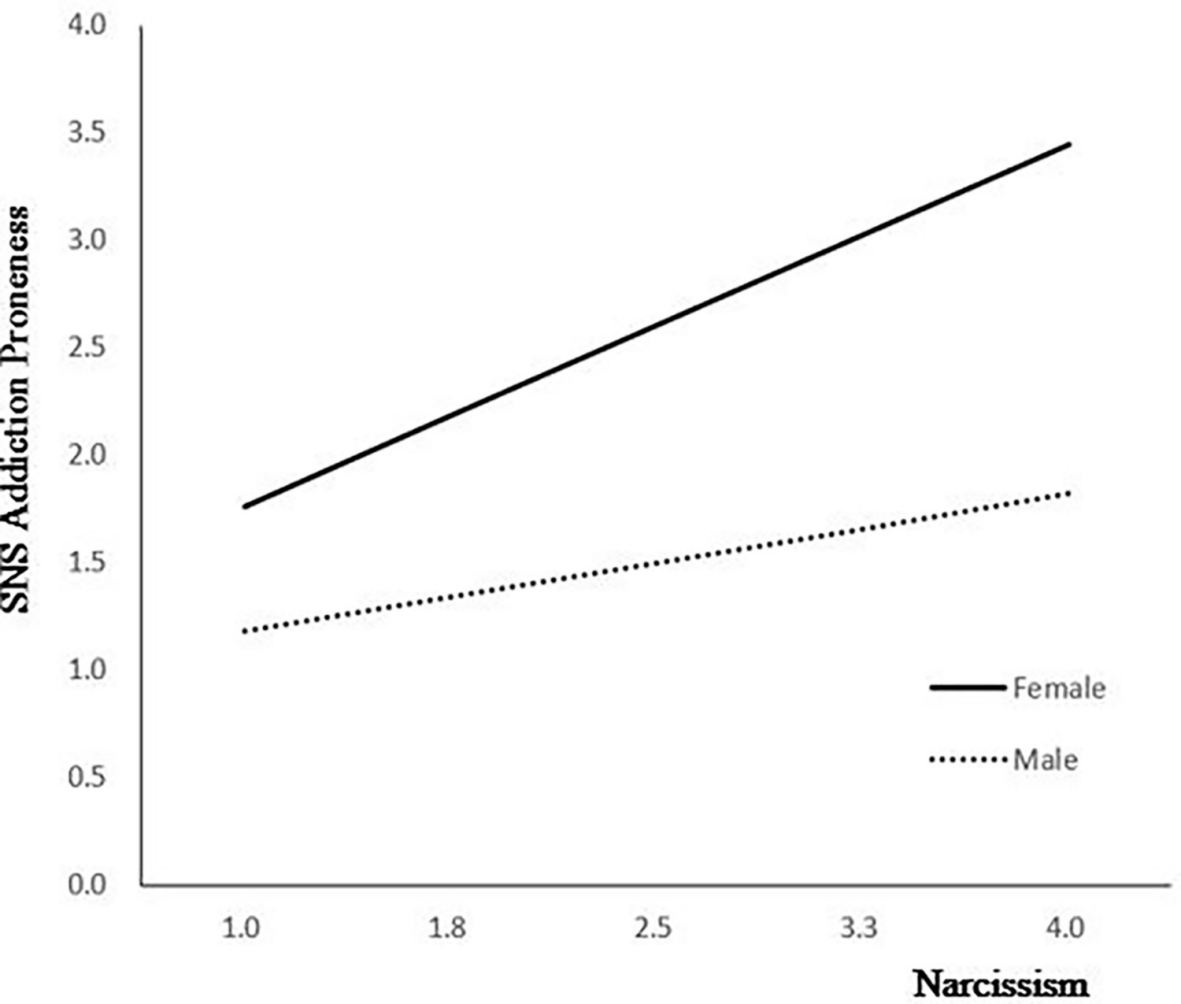
Gender as a moderator of the relationship between narcissism and SNS addiction proneness.

Social anxiety had a significant effect on narcissism in Step 1 (B = 0.14, *p* = .06). In Step 2, the direct effect of social anxiety on SNS addiction proneness was significant (B = 0.21, *p* < .001), but the effect of narcissism on SNS addiction proneness was not significant (B = 0.23 p = .972). The effect of gender on SNS addiction proneness was significant (B = 0.23, p = .002). The effect of the interaction term of narcissism and gender (moderator variable) on SNS addiction proneness was significant (B = 0.35, p = .008). As a result of analyzing the conditional indirect effect of narcissism, it was found that the effect was significant in both male (effect = 0.34, 95% CI = .13~.54) and female groups (effect = 0.69, 95% CI = .53~.85) **(Table 3) (Fig 2)**. The moderated mediation effect was significant in both male and female groups, and the moderated mediation effect was greater in the female group than the male group. For the strength of the mediation effect in which social anxiety affects SNS addiction proneness through narcissism, the positive reinforcement effect increased more in the female group compared to the male group **(Table 3)**.

## Discussions

This study attempts to examine the mediation effect of narcissism and the moderated mediation effect of gender in the effect of social anxiety of university students on SNS addiction proneness.

The results of this study showed that there is a significant difference in social anxiety depending on the percentage of friends on SNS that the participants do not know or have never met offline (in real life). Social anxiety was higher the participants who do not know or have never met offline about 50% of their friends on SNS. SNS enables users to form relationships online and supports various activities such as managing personal connections and sharing information and content, but it rather reduces conversations based on intimate and close relationships with family and friends, thereby deteriorating the quality or depth of interpersonal relationships [23]. Those who have high social anxiety are likely to be extremely nervous or anxious when having a conversation with others or in certain situations, thereby having fear about interpersonal relationships [12]. People with higher anxiety tend to use SNS more as a tool to form social relations, but ‘social’ here is merely superficial, rather than having a negative impact on interpersonal relationships in real life [5,24]. Thus, people with high social anxiety are more active on SNS where they can freely assert and express themselves [6,13]. If most friends on SNS are actually strangers, the relationship is superficial and shallow, thereby experiencing less social anxiety. However, social anxiety increases if 50% are strangers and the other 50% are not.

In this study, there was a significant difference in narcissism depending on personal connections on SNS (friends, followers, group members). Those with around 50 or more personal connections on SNS (friends, followers, group members) had higher levels of narcissism than those with around 30 or fewer. Since users can control whether to disclose or hide their personal information on SNS [25], it may be a space preferred by highly narcissistic people with a strong desire to present themselves perfectly to others [6]. Highly narcissistic people with more personal connections on SNS can receive more positive responses by glamorizing themselves to look positive, thereby satisfying their desire to present themselves perfectly to others. It is necessary to help university students become aware of their own needs that drive them to be so immersed in creating personal connections on SNS. For those who tend to be sensitive to the reactions of others, there is a need to identify whether they internally have narcissism traits such as excessive flattery and ostentatious attitude and apply various strategies accordingly.

The results of this study showed that there was a significant difference in SNS addiction proneness depending on gender, the number of SNS platforms used, average time spent using SNS per day, personal connections on SNS, and how much importance SNS is in daily life. Previous research had also revealed that SNS addiction proneness was significantly higher among female university students, and that those who spend more time using SNS per day had SNS addiction proneness [5]. These results showed that gender and the time spent using SNS had a significant effect on SNS addiction proneness [26]. Those who have more personal connections on SNS and consider SNS more important in daily life also use more SNS platforms and spend more time using SNS per day, thereby resulting in high SNS addiction proneness. Universitys must provide education to prevent SNS addiction proneness for each gender so that students can properly manage their time and have control over the use of SNS.

The results of this study were consistent with previous studies in which social anxiety had a significant positive correlation with narcissism and SNS addiction proneness. Narcissism had a significant positive correlation with SNS addiction proneness [5,13–15]. In other words, this result of social anxiety supports the findings of previous research on the positive relationship between narcissism and SNS addiction proneness, while also confirming that narcissism has a positive correlation with SNS addiction proneness.

Moreover, as a result of examining the mediation effect of narcissism in the effect of social anxiety on SNS addiction proneness, we found that narcissism had a mediation effect. In other words, it was proved that university students with higher social anxiety also have higher SNS addiction proneness, and narcissism aggravated by social anxiety can further increase SNS addiction proneness. These results are in line with previous research revealing that university students with higher narcissism also have higher social anxiety, which leads to higher SNS addiction proneness, although the causality may be different [14,15]. Those who have high social anxiety are likely to be extremely nervous or anxious when having a conversation with others or in certain situations, thereby having fear about interpersonal relationships [12,15]. People with higher anxiety tend to use SNS more as a tool to form social relations, but ‘social’ here is merely superficial, rather than having a negative impact on interpersonal relationships in real life [5,24]. Thus, people with high social anxiety are more active on SNS where they can freely assert and express themselves [6,13,15]. Variables such as social anxiety and narcissism are related to interpersonal relationships [12]. For university students with high social anxiety likely to distort the reactions of others in interpersonal relationships, SNS can be an attractive tool to satisfy their narcissism while avoiding social situations. This supports the result that higher social anxiety leads to higher narcissism, which further increases SNS addiction proneness [12,14,15]. Therefore, by understanding individual tendencies, identifying psychological traits of social anxiety and narcissism, and using individualized strategies accordingly, SNS addiction proneness will be possibly reduced.

In this study, the moderated mediation effect of gender in the effect of social anxiety on SNS addiction proneness through narcissism was significant. In other words, the strength of the mediation effect of social anxiety on SNS addiction proneness through narcissism increased more in the female group. Unfortunately, these findings could not be compared with any previous study since none was found to have investigated the moderated mediation effect of gender, even though some previous studies revealed that there were differences in social anxiety, narcissism, and SNS addiction proneness depending on gender, and that women showed higher social anxiety, narcissism, and SNS addiction proneness than men [5,6,14–16,26,27]. Therefore, it is necessary to develop programs that can mediate social anxiety, narcissism, and SNS addiction proneness of university students together. Furthermore, by implementing interventions to improve interpersonal relationships such as social skills training to reduce social anxiety and narcissism considering gender, it will be possible to reduce SNS addiction proneness of university students.

Most previous studies were focused on the causality between social anxiety, narcissism, and SNS addiction proneness or simple mediation effects. However, this novel study will advanced the understanding of the mediation effect of narcissism and the moderated mediation effect of gender in the effect of social anxiety on SNS addiction proneness of university students and provided in-depth information on the relationship between these variables. Further research can more clearly reveal the causality between variables by using a method that integrates mediation and moderation models rather than analyzing simple mediation or moderation. This study was conducted through convenience sampling of university students in specific regions, and thus there are limitations in generalizing the results. Furthermore, since the variables were measured based on the self-report survey of the participants, the results may be biased, underestimated, or overestimated depending on the subjectivity of the respondents.

## Conclusion

This study examined the mediation effect of narcissism and the moderated mediation effect of gender in the effect of social anxiety of university students on SNS addiction proneness. It revealed that the strength of the mediation effect in which social anxiety affects SNS addiction proneness through narcissism increased more in the female group. Therefore, when developing an intervention program to reduce SNS addiction proneness of university students, it will be more effective to apply strategies to also reduce social anxiety and narcissism of university students simultaneously, as well as programs to improve interpersonal relationships such as social skills training according to gender. The following are suggested based on the results of this study. First, future research should develop customized programs to reduce SNS addiction proneness based on university students’ gender and compare their effects. Second, further studies should be conducted to develop, and implement programs that can reduce social anxiety, narcissism, and SNS addiction proneness simultaneously and validate their effects. Third, as this study has regional limitations, it also suggests a replication study that extends the regions by using revalidated tools.

## Supporting information

S1 File A spreadsheets files containing scripts datasets in this study. (XLSX)
